# A Novel Dog-Bone Oscillating AFM Probe with Thermal Actuation and Piezoresistive Detection ^†^

**DOI:** 10.3390/s141120667

**Published:** 2014-10-31

**Authors:** Zhuang Xiong, Estelle Mairiaux, Benjamin Walter, Marc Faucher, Lionel Buchaillot, Bernard Legrand

**Affiliations:** 1 Institute of Electronic Engineering, China Academy of Engineering Physics, Mianyang 621999, China; 2 Institut d'Electronique, de Microélectronique et de Nanotechnologie—IEMN CNRS UMR8520, NAM6 group, Villeneuve d'Ascq 59650, France; E-Mails: estelle.mairiaux@iemn.univ-lille1.fr (E.M.); benjamin.walter@isen.iemn.univ-lille1.fr (B.W.); marc.faucher@isen.iemn.univ-lille1.fr (M.F.); lionel.buchaillot@iemn.univ-lille1.fr (L.B.); bernard.legrand@isen.iemn.univ-lille1.fr (B.L.)

**Keywords:** AFM, Micromechanical resonator, thermal actuation, piezoresistive detection

## Abstract

In order to effectively increase the resonance frequency and the quality factor of atomic force microscope (AFM) probes, a novel oscillating probe based on a dog-bone shaped MEMS resonator was conceived, designed, fabricated and evaluated. The novel probe with 400 μm in length, 100 μm in width and 5 μm in thickness was enabled to feature MHz resonance frequencies with integrated thermal actuation and piezoresistive detection. Standard silicon micromachining was employed. Both electrical and optical measurements were carried out in air. The resonance frequency and the quality factor of the novel probe were measured to be 5.4 MHz and 4000 respectively, which are much higher than those (about several hundreds of kHz) of commonly used cantilever probes. The probe was mounted onto a commercial AFM set-up through a dedicated probe-holder and circuit board. Topographic images of patterned resist samples were obtained. It is expected that the resonance frequency and the measurement bandwidth of such probes will be further increased by a proper downscaling, thus leading to a significant increase in the scanning speed capability of AFM instruments.

## 1. Introduction

Atomic Force Microscope (AFM) is widely used to characterize surface topography and forces. Since the brilliant demonstration of high-speed (HS) AFM in 2008 by Ando *et al.* [1], the applications of AFM have been widespread in biological research area [2] as well as in materials sciences [3] thanks to the ability to observe dynamic processes. Despite the great success of current HS-AFM systems, challenges such as scanning over larger areas with more appropriate resolution still exist [4]. One key element to achieve the goal should be to further increase the measurement bandwidth by fabricating smaller cantilever probes with higher resonance frequency (*i.e.*, the working frequency of oscillating mode AFM) [5,6]. A previous research work of Kawakatsu *et al.* [7] has shown that the resonance frequency of small cantilevers can reach up to 10 MHz. However, the quality factor of these probes was low, typically *Q* = 5 in air [7], which has a negative impact on the measurement sensitivity. A special designed optical detection system based on Fizeau interferometer was moreover required due to the small size of the cantilevers, which makes it less convenient to realize AFM experiments. Indeed, the laser-based optical detection unit is well known for its high measurement accuracy and it is widely employed in conventional AFM set-ups. However, currently, the incident laser beam should be focused on an area no less than 2 μm in width [5] considering the diffraction limit. Therefore, for these practical considerations, the resonance frequency of AFM cantilevers is hard to exceed 3∼4 MHz in air. Evidences also show that cantilever probes are so sensitive to environmental viscous damping that their quality factors drop abruptly from several hundred in air to lessthan 10 in liquid, which is detrimental to *in vitro* bioimaging applications.

Meanwhile, integrated detection methods such as the piezoresistive [8] and piezoelectric [9] sensing schemes have been developed in purpose of overcoming the constraints of optical detecting method. However, moderate success among HS-AFM users was obtained because of the difficulty of the integrated process at a very small cantilever probe.

To take the advantage of quartz resonators, AFM probes based on quartz tuning forks and length-extension resonators have also been developed successfully [10]. They exhibited excellent temperature-stable properties and high quality factors, and were able to achieve atomic resolution images. Moreover, they could be operated with small oscillation amplitudes because of their high stiffness preventing the tip from snapping into the sample. The main drawback of the quartz material is the low compatibility with batch processing and standard silicon microtechnologies, as a consequence, the dimensions of these probes are thus confined in the millimeter range with limited resonance frequencies around 1 MHz maximum.

In view of that the efforts on modifying the cantilever probes could not effectively prompt the resonance frequency and quality factors, microelectromechanical systems (MEMS) resonators with high resonance frequency and quality factors were considered as promising candidates for AFM probe applications. Bulk mode, especially in-plane vibrating silicon resonators initially developed for RF applications [11–13] appear to be promising candidates as an alternative to AFM cantilever probes for HS AFM applications: (1) in-plane vibration modes suffering less from damping and energy losses than cantilevers' flexural modes due to the minimized interactions with the viscous drag of fluid around; (2) resonance frequencies up to hundreds of MHz, offering a larger measurement bandwidth that would enable time-resolved experiments at the microsecond scale; (3) quality factors exceeding 1000 in air; (4) integrated driving and sensing electromechanical transducers enabling parallel operation, on-chip integration and convenient imaging operation both in air and in liquids.

In the light of the application of MEMS resonators in the field of surface imaging, a new concept of AFM probes exploiting the elliptic mode vibration of a ring shaped resonator with integrated capacitive excitation and detection was proposed by the authors in 2007 [14]. Further research work showed that such AFM probes were capable of surface imaging with a force resolution of several pN/√Hz [15–17]. The resonance frequency of these ring probes reached up to 11 MHz with a quality factor of 1500 in air and the capability of biomolecular imaging was successfully demonstrated [17]. Another advantage of these probes concerns the in-plane nanotip that features an apex less than 10 nm [18], enabling the batch fabricationof ready-to-use AFM probes. The piezoresistive readout was also implemented [19] based on the idea presented by Phan *et al.* [20].

However, the main concern of the ring shaped probe is that the sub-100-nm capacitivetransduction air gap requires large fabrication efforts especially when the ring geometry is shrunk down to several tens of micrometers. The low-order elliptic vibration mode also prevents the devices from reaching higher resonance frequency. For example, the ring probe presented in reference [16] features an air gap of 80nm and a ring radius of 30 μm: the resonance frequency of 11 MHz appears to be the ultimate value for such device. Therefore, an alternative probe design and transduction scheme should be investigated to achieve higher resonance frequencies.

In this paper, a new AFM probe concept based on dog-bone shaped resonator [21] with thermal actuation and piezoresistive detection is proposed, inspired by the works of Bontemps, Beek, Rahafrooz *et al.* [22–24]. The imaging capability of this kind of probes issuccessfully demonstrated. Both modeling and experimental achievements suggest thatdog-bone probes feature simple fabrication process, high resonance frequencies and quality factors, which could be a new option for the the realization of HS-AFM.

## 2. Design and Working Principle

### 2.1. Mechanical Design

**Figure 1.**
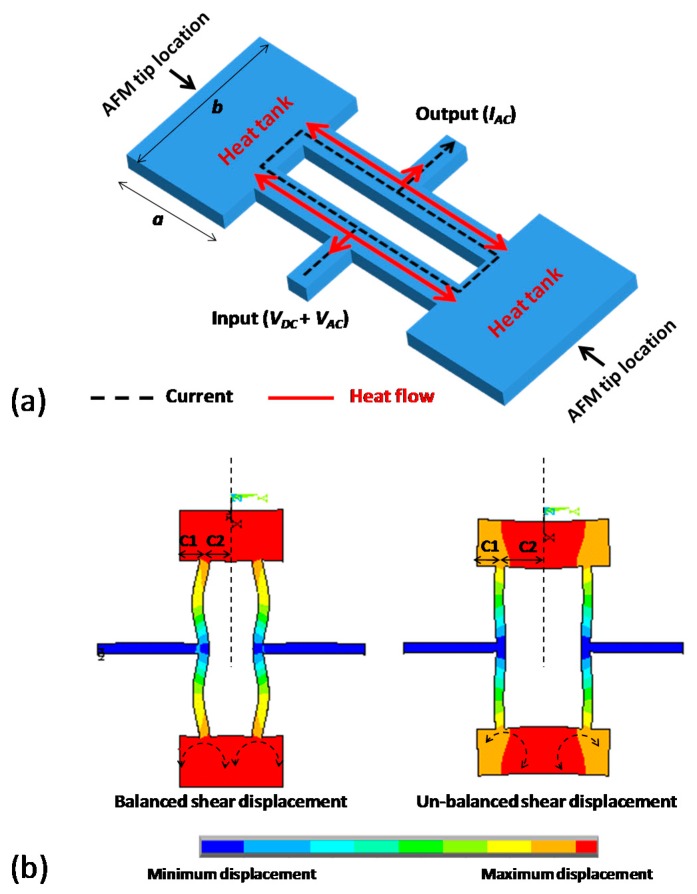
(**a**) Schematic view of dog-bone oscillating probe using thermal actuation and piezoresistive detection; (**b**) Modal analysis of balanced (C1=C2) and un-balanced (C1≠C2) dog-bone structure design. The analysis showed that the balanced design could minimize the shear displacement at the edge of heat tank, and therefore, was the best location for atomic force microscope (AFM) tip positioning.

The working principle of a dog-bone oscillating AFM probe is based on the thermal actuation and the piezoresistive sensing of the mechanical element, which can be described as follows: a time-varying voltage causes a temperature fluctuation in the mechanical structure, leading to an oscillating mechanical expansion/compression; and consequently results in a change of the material electrical resistivity. The dog-bone resonator design mainly consisted of two actuator beams with two connected massive heat tanks (Figure 1a). By applying superimposed DC and AC voltages, temperature fluctuation created inside actuator beams would result in their alternative expansion and compression, leading to a longitudinal vibration of the resonator. As for detection, the time-varying deformation of the actuator beams would cause a periodic change of their electrical resistance. This piezoresistive effect would result in a motional AC current passing through the device and being detected at the output port.

The resonator was anchored at the vibration nodes to minimize energy losses by acoustic radiation [25]. Two tips were symmetrically integrated at the edge of both heat tanks Figure 1a to maintain the structure mechanical balance and eliminate parasitic vibration modes.

In order to obtain the rational lengths of C1 and C2 of heat tank Figure 1b a finite element modeling was carried out. The simulation results showed that a length difference between C1 and C2 might lead to a significant shear displacement at the edge of heat tanks because the un-balanced mass was added along each side of the beam axis. The lengths of C1 and C2, therefore, were taken to be identical in order to minimize the shear displacement at these locations.

**Figure 2.**
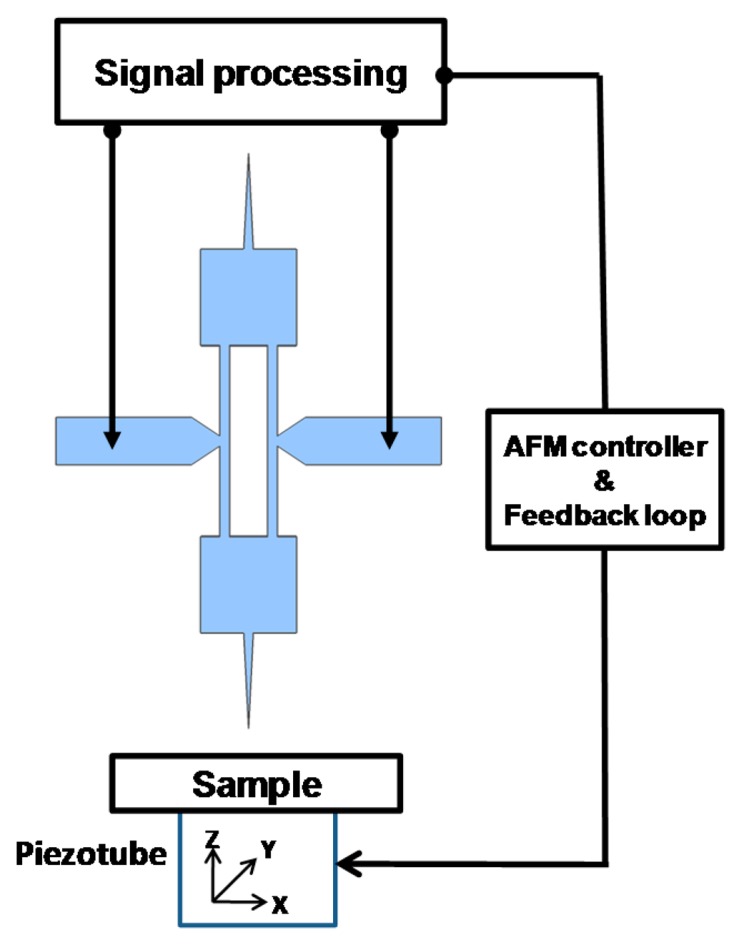
Working principle of AFM microscopy using a dog-bone probe.

Considering the AFM operation, the probe was placed vertically with respect to the sample (Figure 2). When oscillating at a nanometric distance from the surface, the tip was sensitive to short-range forces. The near field interaction force gradient induced a shift in the resonator natural resonance frequency, leading to changes in the probe vibration amplitude and phase. A feedback system (AFM controller) was used to analyze these changes and altered the vertical position (along direction z) of the sample through the piezotube. The displacement required to maintain a constant tip-sample interaction was then interpreted to map the sample surface topography.

### 2.2. Vibrational Characteristics

The assumptions for predicting the vibration characteristics of a free longitudinal vibrating dog-bone resonator (without tips and anchors) applied by Phan *et al.* [26] are used in this paper as well:

1. All components (except the two actuator beams) of the resonator (Figure 1a) are rigid bodies (*i.e.*, no strain appear under any loading).
2. Only the two actuator beams of the resonator (Figure 1a) are elastic bodies (strain occurs only in the two beams).
3. The structure of dog-bone resonator does not undergo significant deformation during vibration.

The equation for predicting the resonance frequency of dog-bone resonator [26] based on the above assumptions is

(1) $$f_{0}=\frac{1}{2\pi }\sqrt{\frac{E}{\rho }}\sqrt{\frac{2w}{ab\frac{L}{2}+[b^{2}+{\left(\frac{L}{2}\right)}^{2}]w}}$$

The equation for predicting the effective stiffness of dog-bone resonator based on the above assumption is considered as the sum of the effective stiffness of two parallel-connected free-free beams:

(2) $$k_{\text{eff}}=\frac{\pi^{2}\text{whE}}{L}$$

At the first stage of the study, the preliminary dog-bone probe geometry was designed large in dimension (compared to the devices presented in [24]) for the sake of fabrication simplicity and AFM manipulation. The resulting structure dimensions are: *L* = 200 μm, *w* = 10 μm, *a* = *b* = 100 μm, h = 2 μm with *E* = 170 GPa, ρ = 2330 kg/m^3^ for silicon. The calculated *k_eff_* and *f_0_* using Equations (1) and (2) were 170 kN/m and 5.5 MHz respectively (ring resonator with similar dimensions would resonate around 1 MHz [15]).

### 2.3. Electromechanical Modeling and Shortcoming OF Direct Measuring Method

The overall transfer function *H_s_(ω)* that describes the relation between input voltage *V_AC_* and output current *I_AC_* can be expressed as [24]:

(3) $$H_{S}\left(\omega \right)=\frac{I_{AC}}{V_{AC}}=-\frac{8\kappa \alpha {V_{DC}}^{2}}{j\omega C_{th}\pi^{2}R_{p}^{2}}{\left(1-\frac{\omega^{2}}{\omega_{0}^{2}}+j\frac{\omega }{Q\omega_{0}}\right)}^{-1}$$

The output piezoresistive current *I_AC_* at resonance frequency can be written as:

(4) $$I_{AC}(\omega )=\frac{8}{\pi^{2}}{I_{DC}}^{2}\frac{\kappa \alpha }{\omega C_{th}}QV_{AC}(\omega )$$

The major drawback of the presented dog-bone resonator is that the thermal driving and the piezoresistive sensing share the same current path. Therefore; the driving signal *V_AC_* also passes directly across the electrical resistance *R_p_* of the silicon structure and results in a feedthrough current *I_AC0_* contributing to the output signal at exactly the same frequency as the piezoresistive current.

(5) $$I_{AC0}(\omega )=\frac{V_{AC}(\omega )}{R_{p}}$$

The total output signal captured at resonance is then the superposition of *I_AC_* and *I_AC0_* with opposite phase because the Gauge factor κ is a negative value considering the n-type silicon. Therefore, an inverted resonance peak which had been confirmed in [27] at resonance frequency was expected to appear in this research. Noticing this, a simple differential measurement method to compensate for the feedthrough current was proposed and introduced in Section 4.2 by the authors.

## 3. Fabrication Process and Results

**Figure 3.**
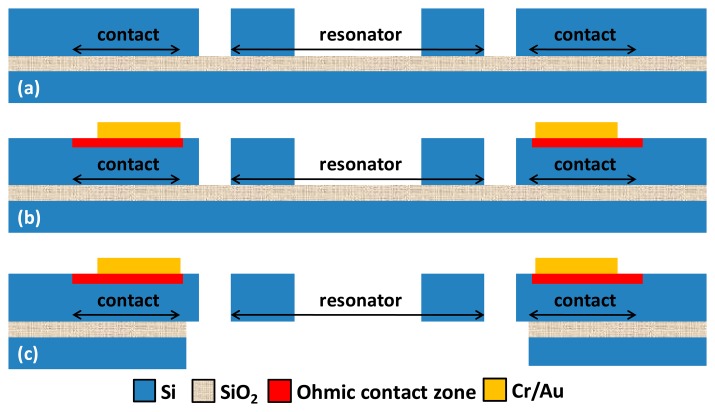
Fabrication process (**a**) Deep Reactive Ion Etching (DRIE); (**b**) Ion implantation and metal deposition; (**c**) DRIE etching and releasing.

The dog-bone AFM probe was fabricated on a n-type SOI wafer (Device layer 2 μm, BOX layer 2 μm, Handle layer 300 μm, doping level 10^18^ atoms/cm^3^) with standard silicon micromachining technology. As illustrated in Figure 3, the resonator layout was first patterned on the device layer by DRIE. Highly doped (10^21^ atoms/cm^3^) zones were then implanted over the surface, intended for ohmic contact. A Cr/Au metal stack was then deposited by evaporation to realize electrical interconnects. The front side of wafer was then protected by a thick resist (SPR220) and another DRIE was processed on the handle layer to create an opening over the backside of resonator. After removing the protection resist, the BOX layer was etched in HF and the resonator was finally released after the supercritical CO_2_ drying process.

**Figure 4.**
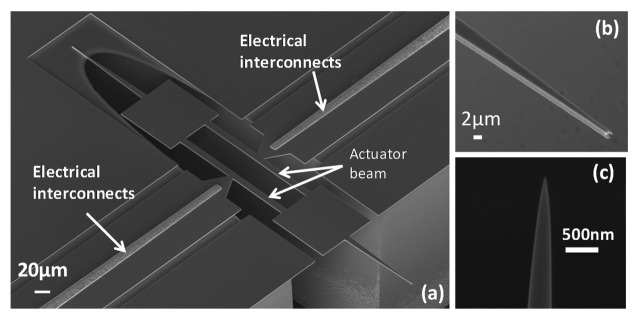
SEM images of (**a**) Overview of fabricated device; (**b**) Close view of the 150 μm long and 10 μm wide massif tip; (**c**) Tip sharpened by Focused Ion Beam etching.

The fabricated device is shown in Figure 4. The actuator beam was 200 μm in length and 10 μm in width. Two symmetric tips were prolonged from the 100 × 100 μm square heat tank. The resonator was held by the trapezoid shape anchors to enhance the support rigidity and facilitate the drain of heat flow from beam to substrate. The electrical accesses (Cr/Au) were placed close to the resonator in order to minimize the ohmic losses from input/output to actuator beams. Considering the fabrication simplicity, the nanotip fabrication step was not integrated for the current version. Therefore, a further FIB milling step was required to obtain a tip apex small enough for AFM operation and satisfying lateral resolution Figure 4c.

## 4. Measurement Methods and Characterizations

### 4.1. Direct Electrical Measurements

**Figure 5.**
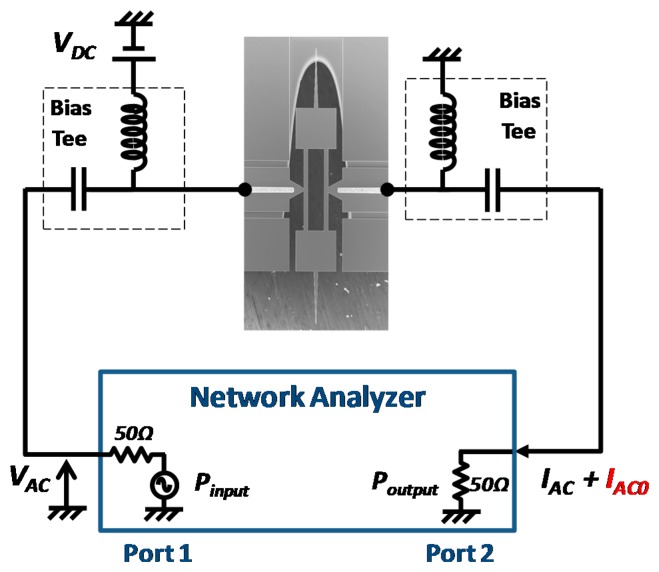
Schematic representation of working principle. *V_DC_* is used to bias the actuator beams for piezoresistive detection. Network analyzer generates a driving signal *V_AC_* and collects the output piezoresistive current *I_AC_*.

Electrical measurements were first carried out to verify the functionality of thermal driving and piezoresistive sensing. Depicted in Figure 5, the DC bias *V_DC_* was applied between anchors to bias the resonator. The network analyzer (VNA) supplied an incident power at port 1 (this power corresponded to an AC excitation voltage *V_AC_* generated over a 50 Ω resistance) which drove the longitudinal vibration of the dog-bone resonator by thermal effect. The change of piezoresistance during the mechanical vibration led to an AC current *I_AC_*, along with the feedthrough current *I_AC0_*, both being collected at port 2 of the VNA.

**Figure 6.**
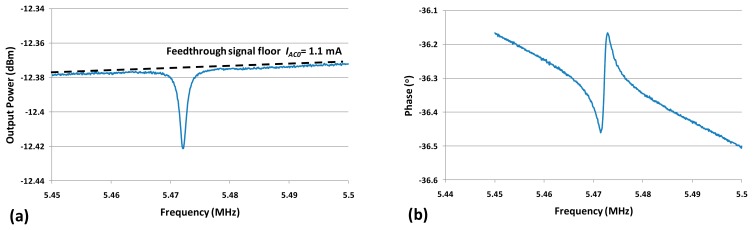
Output signal amplitude (**a**) and phase (**b**) *versus* frequency with input power of 10 dBm (equals to *V_AC_*of 1.4 V). (*V_DC_* = 3 V, Measurement bandwidth = 100 Hz).

The measured resonance frequency of the dog-bone AFM probe was around 5.47 MHz in air with an input power of 10 dBm and a DC bias *V_DC_* = 3 V as shown in Figure 6. The frequency discrepancy between the measured value and the calculated one (5.5 MHz by using Equation (1)) was mainly due to the additional mass of AFM tip. Other sources such as the temperature dependent Young's modulus or dimensions changes during fabrication process (such as lithography, DRIE.) could also cause a slight frequency shift between measurement and calculationresults.

The resonance peak was inverted and superimposed with a feedthrough current *I_AC0_*, as explained in Section 2.3. By converting the output power into current over the 50 Ω load of the VNA port, the corresponding *I_AC0_* could be obtained (shown in Figure 6). The device electrical resistance *R_p_* was thus the quotient of *V_AC_* and *I_AC0_* which was about 1.3 kΩ.

**Figure 7.**
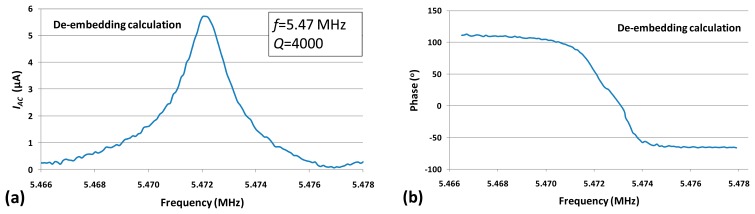
De-embedded piezoresistive current *I_AC_* (**a**) and phase (**b**) *versus* frequency with input power of 10 dBm. (*V_DC_* = 3 V, Measurement bandwidth = 100 Hz).

In order to obtain the resonance characteristic from the electrical measurement, the device signal was de-embedded (by vectorial subtraction of the feedthrough signal floor from the real and imaginary part of the device output signal), the data was further processed, and the purely motional response was extracted (Figure 7).

The quality factor of the device was measured to be about 4000 in air as indicated in Figure 7. Experimentally, it would be possible to retrieve purely resonance peak by using a frequency mixing technique or a two-port balanced measurement with an inactive resonator [28]. However, these techniques require either extra signal processing circuits or extra fabrication efforts. In this study, a simple differential measurement method to eliminate the feedthrough signal was developed and introduced in the following section.

### 4.2. Differential Electrical Measurements

**Figure 8.**
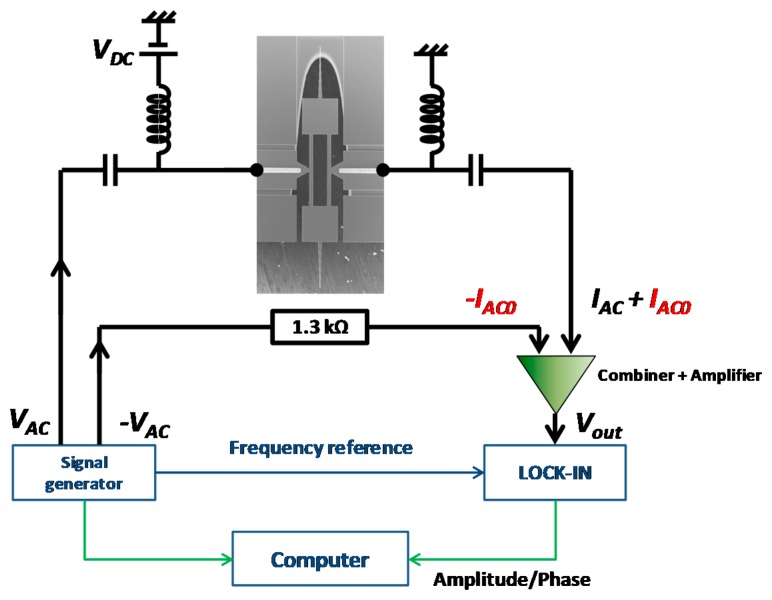
Schematic representation of the differential measurement to compensate *I_AC0_*.

In order to effectively eliminate the feedthrough signal, a differential measurement system was designed. The operation principle of differential measurement is shown in Figure 8. The signal generator supplies two AC signals with same amplitude but opposite phase (±*V_AC_*). *V_AC_* and *V_DC_* drive the resonator and cause a superposition of *I_AC_* and *I_AC0_*. −*V_AC_* passes across a resistor of the same electrical resistance *R_p_* as the one of the resonator, and therefore, creates -*I_AC0_*. By adding the output currents from both two branches, *I_AC0_* is thus cancelled. The whole system is computer controlled which set the frequency of the source of the signal generator and acquires the corresponding vibration magnitude and phase information from the lock-in amplifier.

**Figure 9.**
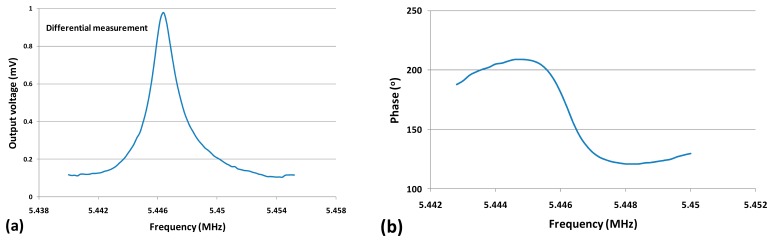
Output voltage (**a**) and phase (**b**) *versus* frequency of the differential measurement with *V_AC_* = 0.9 V. (*V_DC_* = 3V, Measurement bandwidth = 100 Hz).

An experimental study was performed to verify the effect of the proposed differential measurement method. One of the experimental results is shown in Figure 9, indicating the output voltage and phase *versus* frequency of the differential measurement with *V_AC_* = 0.9V. It can be seen that as the feedthrough signal was effectively eliminated, the clear resonance peak and phase rotation were observed. The signal to noise ratio measured around resonance frequency was about 1000 √Hz with a quality factor about 4000.

## 5. AFM Imaging

### 5.1. Characterization of the Vibration Amplitude

**Figure 10.**
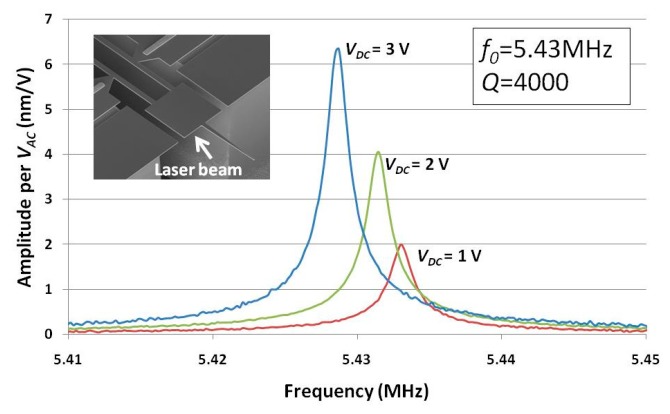
In-plane vibration amplitude (nm/*V_AC_*) at tip location *versus* frequency with different bias voltage *V_DC_*.

Prior to AFM imaging experiments, the mechanical amplitude of the dog-bone resonator at tip location was measured as it would be further used to calibrate the AFM operation parameters and to estimate the minimal detectable force. The measurement was carried out in air with a Polytec MSA 500 laser vibrometer. The laser beam was focused at the outer edge of heat tank of which the vibration amplitude was the same as that of the probe tip. The vibration amplitude *versus* frequency curves with different bias voltage *V_DC_* of 3V, 2V, and 1V are shown in Figure 10. The corresponding vibration amplitudes were 6.2 nm, 4 nm and 2 nm per *V_AC_* = 1 V respectively. The resonance frequency was slightly shifted to lower frequencies as *V_DC_* increased due to the device self heating and the temperature dependent Young's modulus [29]. The quality factor measured was about 4000, which was consistent with the former electrical measurement (refer to Section 4.1).

### 5.2. Experimental AFM Set-Up

**Figure 11.**
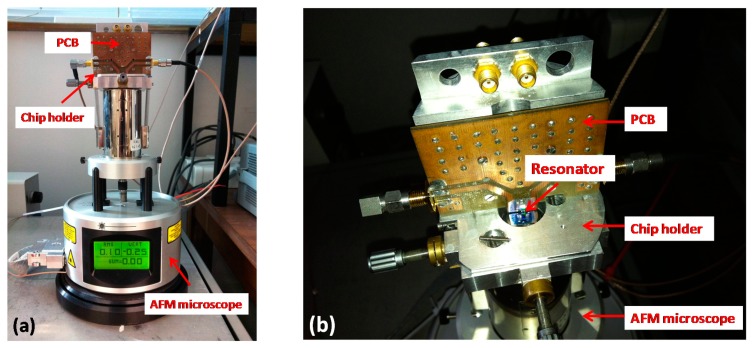
Experimental set-up for AFM imaging. The probe was mounted onto a commercial AFM microscope by using a dedicated printed circuit board (PCB) and a rigid chip holder. (**a**) Overview of the AFM experimental set-up; (**b**) Close view of the AFM head.

The dog-bone probe was then mounted onto a commercial AFM set-up with a rigid chip holder (Figure 11). The interconnections between dog-bone probe and the AFM control devices were established on a printed circuit board (PCB). The AFM set-up mainly consisted of a piezoelectric scanner (Multimode, Bruker) for sample positioning and a Nanonis controller (Nanonis, SPECS) dedicated to signal acquisition and feedback loop control. The microscope was placed on an anti-vibration table to minimize the external mechanical perturbations during the scanning.

### 5.3. Approach-Retract Curves

**Figure 12.**
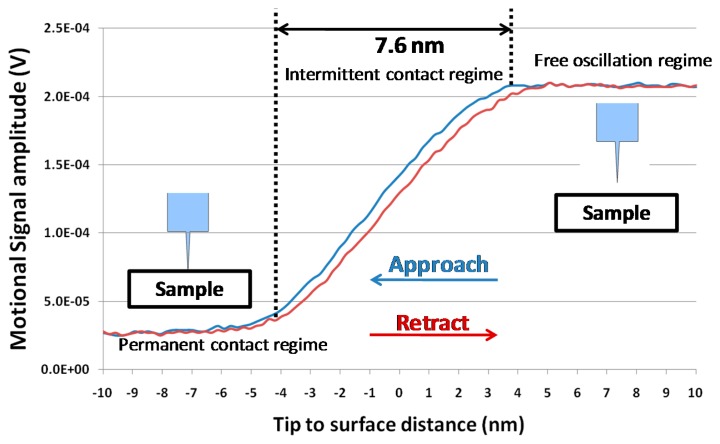
Demodulated signal amplitude as a function of the tip-to-surface distance. The probe was driven by a 3 V DC voltage and 1.23 V AC voltage at 5.43 MHz.

Approach-retract curves represent the vibration amplitude signal of the probe resonator at a given driving frequency as a function of the tip-to-surface distance. The device electrical response was measured when its tip was approached to and retracted from the surface thanks to piezoelectric scanner. In such experimental conditions, an interaction force (dependence of the tip-to-surface distance) acted on the probe tip. Figure 12 presents the magnitude of the resonator output signal as a function of the tip-to-surface distance, for a driving frequency corresponding to the probe resonance frequency. The DC bias was set to 3 V with a 1.23 V AC driving voltage. The signal amplitude was almost constant when the tip was far from the surface, and the signal amplitude was decreased when the tip entered into the intermittent contact regime with the surface (Figure 12). Evidence indicated that the natural resonance frequency shifted to higher frequencies due to the repulsive interaction and consequently the tip vibration amplitude decreased. When the probe was in permanent contact with the surface, there was no more mechanical oscillation of the resonator. The free oscillation amplitude could also be estimated by measuring the extension of the intermittent contact regime (7.6 nm) that was consistent with the measurement obtained by laser vibrometry (Figure 10) for the DC and AC voltages used to drive the resonator.

### 5.4. Sample Surface Imaging

**Figure 13.**
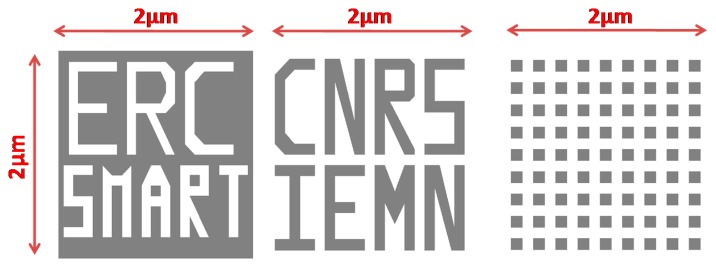
Schematic of the 2 × 2 μm square Polymethyl Methacrylate (PMMA) sample designed for surface imaging. The depth of the patterns was about 100 nm (dark color).

A total of three samples for investigation were made by patterning Polymethyl Methacrylate resist (PMMA) over a silicon substrate (Figure 13). The dimensions of each sample were 2 × 2 μm square with a feature depth of 100 nm (dark color). The smallest feature profile was a 100 nm wide square. The AFM working mode being used in this study was amplitude modulation with fixed driving voltage and frequency.

**Figure 14.**
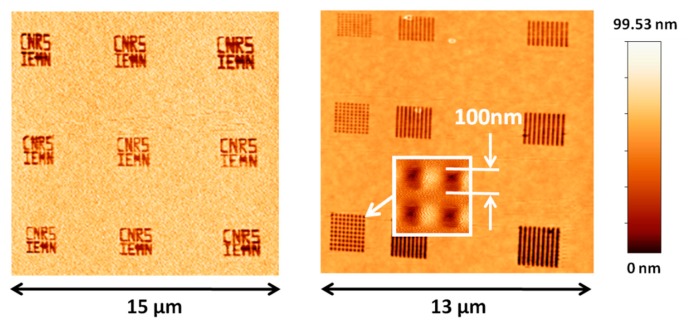
Surface imaging obtained by the dog-bone probe working at 5.4 MHz. The tip velocity was 10 μm/s. Acquisition time = 25 min. (*V_DC_* = 3 V, *V_AC_* = 1.23 V).

Figure 14 shows the overview of some of the patterns obtained by AFM imaging. Each acquired square image contained 1024 × 1024 pixels and the tip velocity was 10 μm/s. The pattern average depth measured was 99.53 nm, nearly exactly matching the practical sample thickness of 100 nm. The smallest square motifs featuring a width of 100 nm were clearly imaged (Figure 14).

**Figure 15.**
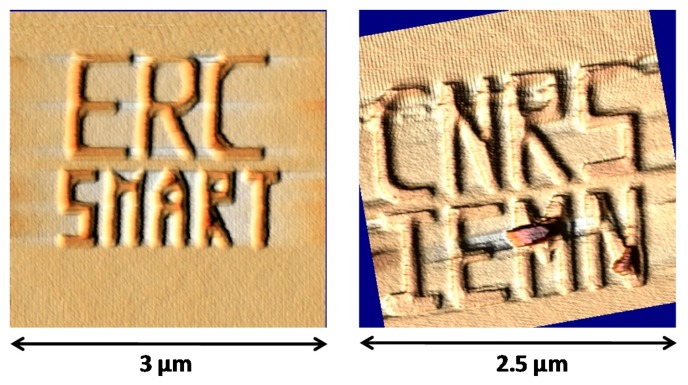
Sample surface AFM topographies obtained by the dog-bone probe working at 5.43 MHz with a tip velocity of 10 μm/s and an acquisition time of 10 min. (*V_DC_* = 3 V, *V_AC_* = 1.23 V).

Figure 15 shows two images acquired over a 2 × 2 μm sample pattern. Both images contained 512 × 512 pixels and the tip velocity was 20 μm/s. The topography was clearly defined with a resolution allowing distinguishing sample patterns and some surface defects (letters “M” and “N”).

### 5.5. Future Improvement on Resonance Frequency and Quality Factor

The major advantages of the proposed dog-bone probe are simple fabrication process, high quality factor and good structure flexibility for reaching higher resonance frequencies. The current dimensions (total 400 μm in length and 100 μm in width) and simple fabrication process enable the dog-bone probe to be downscaled significantly in the future. The resonance frequency of the future miniaturized probes, therefore, is hopeful to reach 100 MHz, much higher than those of ring probe and cantilever probe in the same dimension scale. On the other hand, the high quality factor could also remarkably improve the imaging performances in liquid. Future work also consists of in-liquids experiment where the probe would be coated by a thin isolation layer such as silicon nitride or parylen. It is expected that dog-bone probe would be then able to realize high speed/quality AFM images both in air and liquid and achieve the goal of *in situ* imaging of live cells.

## 6. Assessment of Probe Resolution

### 6.1. Force Resolution

**Figure 16.**
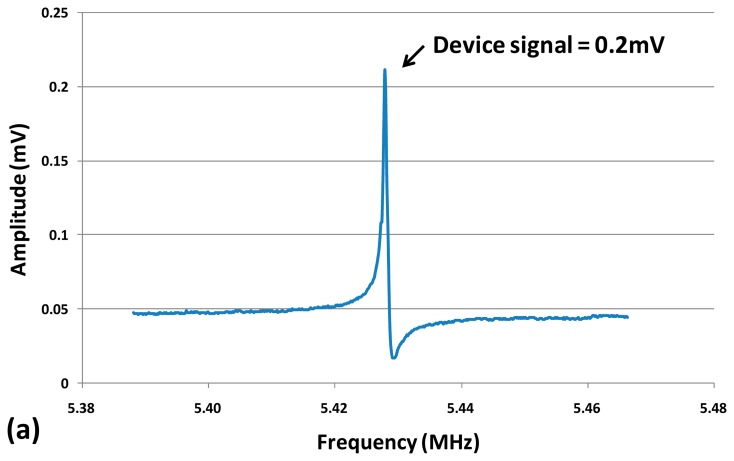

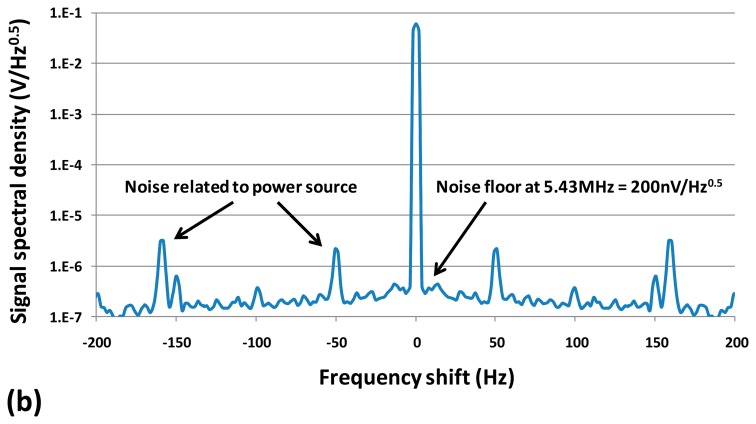
Frequency response (**a**) and spectral density (**b**) of the output signal of the 5.43 MHz dog-bone probe measured by a Nanonis controller. The resulting SNR is 1000 √Hz (*V_DC_* = 3 V, *V_AC_* = 1.23 V).

The output signal amplitude and spectral density of the dog-bone probe are shown in Figure 16 based on characterization results. The signal-to-noise ratio (SNR) was deduced to be 1000 √Hz by calculating the ratio of device signal over noise floor signal around resonance frequency. Minimal detectable vibration amplitude was accordingly obtained based on the SNR value. For the current measurement conditions (*V_DC_* = 3 V, *V_AC_* = 1.23 V), the vibration amplitude (*A_0_*) at tip location was 7.6 nm.

The minimal detectable amplitude of the dog-bone probe was then given by:

(6) $$A_{\min}=\frac{A_{0}}{\text{SNR}}\approx 7.6\text{pm}/\sqrt{\text{Hz}}$$

Assuming a constant force gradient, the minimal detectable force could be therefore derived using

(7) $$F_{\min}=\frac{k_{\text{eff}}}{Q}A_{\min}$$

The calculated minimal detectable force and the mechanical properties of the dog-bone probe are presented in Table 1.

As indicated in Table 1, the force sensitivity of the current developed dog-bone probe only reaches 320 pN/√Hz (Table 1), which is two orders of magnitude lower than those of ring probe (10∼20 pN/√Hz [19]) and commercial cantilever probes (several pN/√Hz). In the following section, the tactics for significantly prompting force sensitivity will be discussed.

### 6.2. Noise Sources

**Figure 17.**
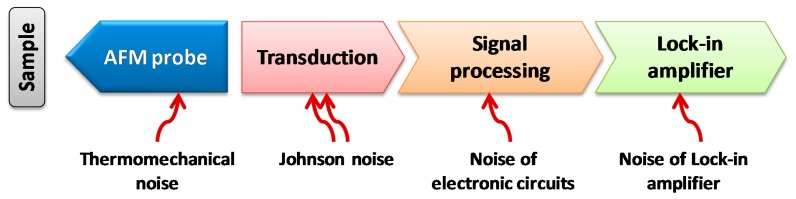
Noise sources identified for the current AFM imaging system.

To significantly prompt the force resolution of the dog-bone AFM probe, noise sources were identified. In general, the noise sources of such AFM system Figure 17 were identified as: (1) the thermomechanical noise at room temperature imposed by the structure material and geometry; (2) the Johnson noise related to the piezoresistance; (3) the electrical noise added by the signal processing electronics; (4) the electrical noise added by the lock-in amplifier.

The measured minimum detectable force (320 pN/√Hz) is the total noise contributed by all noise sources. To facilitate the comparison, each noise sources was thus normalized into a corresponding force noise. For example: (1) the thermomechanical noise calculation gave the noise in force directly [1]; (2) the Johnson noise was represented by an output noise current and the equivalent noise in force was calculated based on the transfer function that describes the relation between vibration amplitude and the output piezoresistive current [24]; (3) the noise of lock-in amplifier was measured about 15 nV/√Hz under given conditions (*V_DC_* = 3 V, *V_AC_* = 1.23 V) around resonance frequency and the equivalent noise in force was 25 pN/√Hz; (4) the noise of signal processing electronics was deduced by subtracting other sub noise sources from the total noise value. Based on the above tactics, the values of different noise sources are estimated and shown in Table 2.

According to Table 2, the thermomechanical noise and Johnson noise are negligible compared to the signal processing electronics. Therefore, the future research should be mainly focused on improving the signal processing electronics in terms of noise floor.

Another way to reduce the signal processing noise level is to increase the piezoresistive transduction efficiency. A drawback of the current dog-bone probe is that the fabrication is based on uniformly doped silicon. Such feature is against the piezoresistive design concept and reduces the transduction efficiency. It is expected that using locally doped piezoresistors at maximum strain location on the actuator beams will be beneficial to force sensitivity. Once the piezoresistive transduction efficiency and the signal processing electronics are successfully optimized, the noise at the input of lock-in would effectively be reduced simultaneously.

## 7. Conclusions

A novel AFM probe based on an in-plane oscillating dog-bone resonator was conceived, designed, fabricated and evaluated. Within the indicated scope of this study, the following conclusions may be arrived at:

(1) The resonance frequency of the fabricated device is 5.43 MHz, which is remarkably higher than those of existing cantilever probes. The current large dimensions and simple fabrication process allow the probe to be further miniaturized without excessive fabrication effort, and consequently to reach significantly higher resonance frequencies.
(2) The quality factor of the fabricated device is 4000 in air, which is at least 10 times higher than those of existing cantilever probes.
(3) The minimum detectable force of 320 pN/√Hz of the current developed probe is at least two orders of magnitude higher than expected for high-performance AFM probes. Further research on optimizing the signal processing electronics and increasing the piezoresistive transduction efficiencyisneeded to achieve a force resolution in the pN/√Hz range.

## Figures and Tables

**Table 1.** Minimal detectable force *F_min_* deduced for dog-bone AFM probe.

**Probe Dimensions**	***L* = 200 μm, *w* = 10 μm *a* = *b* = 100 μm, *h* = 2 μm**
*f_0_* (MHz)	5.4
*k_eff_* (N/m)	1.7 × 10^5^
*Q*	4000
*A_min_* (pm/√Hz)	7.6
*F_min_* (pN/√Hz)	320

**Table 2.** Estimation of the values of the different noise sources.

**Noise Type**	**Noise Source**	**Value(pN/√Hz)**
Thermomechanical	Structure vibration	0.14
Johnson noise	Piezoresistor	0.04
Electrical 1	Signal processing electronics	318
Electrical 2	Lock-in amplifier	25

## Notations

*L*: Actuator beam length
*w*: Actuator beam width
*h*: Resonator thickness
*a*: Heat tank length
*b*: Heat tank width
*f_0_*: Resonance frequency
*k_eff_*: Effective stiffness
*m_eff_*: Effective mass
*Q*: Quality factor
*E*: Young's modulus of silicon
ρ: Mass density of silicon
*R_p_*: Structure electrical resistance
*C_th_*: Thermal capacitance of actuator beams
α: Thermal expansion coefficient of silicon
κ: Gauge factor of n-type silicon
*V_DC_*: DC bias voltage
*V_AC_*: AC driving voltage (RMS)
*I_DC_*: DC bias current
*I_AC_*: Motional current (AC)
*I_AC0_*: Feedthrough current (AC)
*A_min_*: Minimum detectable vibration amplitude
*F_min_*: Minimum detectable force
